# Students’ Occupational Aspirations: Can Family Relationships Account for Differences Between Immigrant and Socioeconomic Groups?

**DOI:** 10.1111/cdev.13378

**Published:** 2020-06-23

**Authors:** Stephanie M. Plenty, Jan O. Jonsson

**Affiliations:** ^1^ Institute for Future Studies (IFFS); ^2^ Swedish Institute for Social Research, Stockholm University; ^3^ Nuffield College, Oxford University

## Abstract

Immigrant background and disadvantaged socioeconomic background are two key predictors of poorer school achievement in Europe. However, the former is associated with higher while the latter is associated with lower aspirations. This study asks whether family relationships account for this difference. Data come from 5,926 students in Germany and Sweden, eliciting indicators of family background and relationships at age 14–15 years (2011) and occupational aspirations 1 year later. High aspirations were found among students of non‐European background and students with higher parental occupational status. Structural equation models showed that while immigrant families had greater parental aspirations and encouragement, family cohesion, and parental monitoring, only parental aspirations mediated the effects of family background.

Students of lower socioeconomic background and students of immigrant background are often identified as educationally disadvantaged because they typically—although not systematically—share in poorer school achievement, as measured by grades or test results (Heath & Brinbaum, 2014). However, in many countries, these two dimensions of family background have radically different associations with educational attainment. Socioeconomically disadvantaged students (e.g., lower parental education or occupational status) make, on average, less ambitious educational choices at given levels of performance than children from more privileged backgrounds (e.g., Jackson, 2013). Contrary to that, children of immigrants typically make *more* ambitious choices at given achievement levels and socioeconomic background than their non‐immigrant peers (e.g., Jackson, Jonsson, & Rudolphi, 2012).

How can we account for this difference in educational choices according to immigrant and socioeconomic background? What is it about immigrant families that compensate for lower grades and instead promote aspirations? This study focuses on family relationships as a potential mechanism for making sense of this empirical anomaly, and occupational aspirations as an indicator of “immigrant optimism” (Kao & Tienda, 1995) that drives higher educational attainment. In studies of educational stratification, it is commonly believed that non‐material parental support is a key mechanism behind sociodemographic group differences in youth’s educational outcomes. Psychological and sociological perspectives emphasize the importance of non‐material support in term of parents’ attitudes and behaviors in shaping their children’s achievement‐related attitudes, and how youth perceive school and their own opportunities for the future (e.g., Brooks‐Gunn & Markman, 2005; Coleman, 1988; Eccles & Harold, 1996; Erikson & Jonsson, 1996). The fact that academic values, familism, and monitoring of behavior is often stronger in many immigrant groups (Fuligni, Tseng, & Lam, 1999; Ghazarian, Supple, & Plunkett, 2008), suggests that non‐material parental support in terms of family relationships may play a vital role in explaining the high aspirations of immigrant youth.

Drawing on nationally representative, longitudinal data from secondary school students in two European countries, this study analyses family relationships as a mechanism that may explain the diverging influence of immigrant background and socioeconomic status on children’s aspirations. Structural Equation Modeling (SEM) is utilized to examine the role of four aspects of family relationships: parental aspirations, parental encouragement, family cohesion, and parental monitoring. Our main aim is to test whether these aspects of family relationships are stronger among immigrant families and are positively associated with students’ aspirations, thereby accounting for the “immigrant optimism” phenomenon.

## Conceptualizing Youth’s Aspirations

Occupational and educational aspirations are important because they reflect students’ school‐related values, intentions and desires as well as goal‐directed behavior. During adolescence, youth begin to consider their future and make choices according to their ambitions, which gradually become more refined (Gottfredson, 1981; Nurmi, 2004). This choice process reflects not only socialization and “pure” interest but also perceived and actual opportunities for reaching one’s goals, which are affected by family socioeconomic and ethnic characteristics (Erikson & Jonsson, 1996). Aspirations are theoretically distinct from expectations, with the latter referring to anticipated outcomes in consideration of restrictions. This study examines aspirations because we are primarily interested in students’ motivations and hopes regarding their future. Although aspirations may not always be fully realized, they are a good predictor of later educational and career attainment (Ashby & Schoon, 2010; Beal & Crockett, 2010). As aspirations are considered one of the key proximate mechanisms behind educational and occupational inequality (Sewell & Hauser, 1980), understanding their origins is important for understanding sociodemographic group differences in educational and occupational pathways.

## The Role of Family Relationships in Understanding Family Background Differences in Youth’s Aspirations

Stratification research often studies differences in student aspirations and attainments in terms of socioeconomic circumstances, observing that students from socioeconomically advantaged families tend to have higher aspirations than socioeconomically disadvantaged students (Jackson, 2013). However, there is widespread recognition that non‐material parental support is critical in shaping students’ ambitions, choices, and subsequent educational attainment. Such support is often conceptualized as parental involvement, and crucially comprises family relationships reflecting a range of parent–child interactions, parenting strategies, and relationship dynamics (see Kurtz‐Costes, 2015; Spera, 2005 for reviews). For example, parents of higher socioeconomic status tend to be more involved in learning activities, hold higher aspirations, and discuss educational and career pathways more than socioeconomically disadvantaged parents (Carolan & Wasserman, 2015; Cheadle & Amato, 2011; Hill & Taylor, 2004; Roska & Potter, 2011). Such families are also characterized by relationships of higher cohesion, or by greater monitoring of children’s whereabouts and behavior (Conger et al., 2002; Duncan & Magnuson, 2003; Melby, Conger, Fang, Wickrama, & Conger, 2008). In Coleman’s (1988) theory of social capital, the transmission of parental human capital to children relies precisely on the relationship quality between parents and children.

Higher aspirations are often also observed among immigrant groups compared to the majority population (e.g., Dollmann, 2017; Jackson et al., 2012; Kao & Tienda, 1995). Although there is a variation between subgroups (Jonsson & Rudolphi 2011; Heath & Brinbaum, 2014), immigrant parents tend in general to hold particularly high hopes for their children’s future (Feliciano & Lanuza, 2016; Raliegh & Kao, 2010) and may consider education as a strategy through which such ambitions can be realized, thus communicating a high value placed on school engagement to their children. This may be especially so as many of them are drawn from the upper part of the educational distribution in their country of origin but have experienced blocked opportunities in the host country (e.g., Engzell, 2019; Feliciano & Lanuza, 2017).

High aspirations of immigrant students may also emerge because of greater “familism” characterizing many immigrant groups (Parasnis & Swan, 2017; Schwartz et al., 2010), which may increase the likelihood that parents’ own aspirations for their children are transmitted (Tjaden & Hunkler, 2017). Indeed, immigrant families in Europe do have stronger family bonds than non‐immigrant families, which contributes to immigrant youth’s mental health advantage (Mood, Jonsson, & Låftman, 2017). Alternatively, higher aspirations and educational attainment might also be explained by some immigrant groups coming from cultures with greater emphasis on educational and work discipline as well as parental authority and monitoring (Lauglo, 2000; Rosenbaum & Rochford, 2008). It is possible that stronger family ties and a greater disciplinary focus among immigrant families provide important sources of support and guidance for students’ ambitions.

It appears then that parental educational involvement, parental monitoring, and family cohesion are features of family relationships that are relevant to understanding socioeconomic and ethnic patterning of youth’s aspirations. Generally speaking, parental educational involvement refers to parental practices and beliefs related to schooling that encourage a child’s academic success (Eccles & Harold, 1996; Hill et al., 2004). Although it involves multiple components, attitudes, and behaviors reflecting parental expectations, values and support (also discussed in terms of “academic socialization”) have been identified as more consistently related to student outcomes than many other types parental of involvement (Boonk, Gijselaers, Ritzen, & Brand‐Gruwel, 2018; Castro et al., 2015; Hill & Tyson, 2009). Socializing children about the importance of education involves communicating attitudes and expectations about the value of learning and succeeding in school, and providing support and motivation to do so. It is theorized to promote academic attainment by shaping students’ own aspirations, expectancies, values, and behaviors (Eccles & Harold, 1996; Wigfield, Byrnes, & Eccles, 2006). Previous studies have found that parental expectations and aspirations, as well as encouragement and support, predict children’s future aspirations, grades and educational attainment, beyond the effects of previous achievement and sociodemographic factors, such as parental education, family economy, or ethnicity (Benner, Boyle, & Sadler, 2016; Hill et al., 2004; Jeynes, 2007; Muller, 1993; von Otter, 2014, see Boonk et al., 2018 for a review).

Despite not necessarily explicitly targeting schooling, processes relating to household rules, parenting style, family communication, and social atmosphere are also aspects of family relationships that are associated with school‐related outcomes (Jeynes, 2010, 2018; Lansford et al., 2018; Steinberg, Lamborn, Dornbusch, & Darling, 1992). Various motivational theories draw on concepts such as belongingness, attachment, and relatedness—all related to family cohesion—to argue that positive family relationships function as a source of emotional security and support, from which students’ competency beliefs and goals can develop (Furrer & Skinner, 2003 for a review). Hill and Wang (2015) demonstrated this link empirically, finding that students who reported greater trust, togetherness, fun, and warmth in their families had higher future educational attainment.

Parental monitoring, on the other hand, involves providing a structured home environment, while demonstrating clear and consistent behavioral expectations and awareness of children’s whereabouts and behavior (Stattin & Kerr, 2000). This parenting practice has in some studies been linked with greater aspirations and positive school outcomes among secondary students (Hill & Wang, 2015; Lowe & Dotterer, 2013), presumably due to parents’ consistent behavioral expectations promoting more self‐discipline, school orientation, and time spent on schoolwork.

## Interrelations Among Family Background, Family Relationships, and Youth’s Aspirations

As non‐material parental resources appear to be weaker for children of lower socioeconomic background but higher for those of immigrant background, family processes such as parental educational involvement, familism, and behavioral monitoring can be expected to mediate the effects of socioeconomic and immigrant background on students’ own aspirations. Although this pathway is often assumed, it has surprisingly seldom been tested. von Otter (2014) found that parental educational aspirations accounted for 16% of the association between family socioeconomic resources and educational attainment in middle age. Carolan and Wasserman (2015) found no mediation effects of parental expectations, concerted cultivation (measured as encouraging activities and discussing school), or school‐based activities on the relationship between mother’s education and their child’s later grade point average. However, as few studies have investigated the extent to which different aspects of family relationships mediate the link between family background and student aspirations or attainment, evidence for (or against) this pathway is lacking. Thus, it is of interest to analyse whether stronger family relationships among immigrant families and among socioeconomically advantaged families can explain why immigrant background but also higher socioeconomic background predict higher aspirations.

If family relationships are a key mechanism through which parental human capital influences student outcomes, *ceteris paribus*, improving these family processes in disadvantaged groups will equalize educational aspirations and attainment. However, this potential also depends on whether family relationships are equally influential in different sociodemographic groups. If the positive effects of family relationships require familiarity with the educational system, for example (e.g., Bourdieu & Passeron, 1977), stronger family relationships may not benefit children of disadvantaged origins. On the other hand, it is possible that students from disadvantaged groups are more sensitive to positive influences due to their otherwise precarious situation (cf. Kao & Tienda, 1995), and therefore the positive effects of family relationships on students’ aspirations might be stronger for students with an immigrant or lower socioeconomic background than for sociodemographically advantaged youth (Wang & Sheikh‐Khalil, 2014).

To date, the empirical findings on how parental educational involvement and other aspects of family relationships interact with socioeconomic and ethnic background are inconclusive (Hill & Tyson, 2009). Hill et al. (2004) and von Otter and Stenberg (2015) found support for an interaction effect that favored students of disadvantaged origins, whereas Benner, Graham, and Mistry (2008) and McNeal (1999) found the opposite; yet, other studies observe no interactions between family background and parental involvement on educational and occupational outcomes (e.g., Arens & Jude, 2017; Jeynes, 2005, 2007; Ream & Palardy, 2008). However, none of these studies explicitly examined the simultaneous importance of students’ socioeconomic and immigrant background.

## The Role of Family Relationships Across European Countries

The “immigrant optimism” phenomenon is observed in the United States (Kao & Tienda, 1995) and across European countries (e.g., Dollmann, 2017; Heath & Brinbaum, 2014; Jackson et al., 2012). While the generality of family processes is our focus and the role of family relationships in students’ aspirations is likely to be similar across Western societies, it is important to also consider variation between our countries. Germany and Sweden differ somewhat when it comes to specific immigrant origin countries and Sweden has slightly more recent (first generation) immigrants than Germany (Jonsson, 2018), which may affect the importance of family relationships. However, the regional background of the immigrant groups is rather similar, with both countries having a large share from MENA (Middle East/North Africa) origins.

However, a prominent difference between Germany and Sweden that is likely to affect occupational aspirations is the school system and its relation to the labor market. Sweden has a comprehensive school system with mixed‐ability classes, while Germany has a famously early‐tracked system (Blossfeld, Buchholz, Skopek, & Triventi, 2016). The benefits of family relationships for aspirations may be weaker in Germany where, at age 14–15, students’ career pathways are already steered (particularly in the lower tracks), compared to Sweden where students’ options remain unrestricted. On the other hand, in Germany educational qualifications tend to be more important for labor market outcomes than in Sweden (e.g., Shavit & Müller, 1998), so parental aspirations and other parental efforts captured by family relationships may be more influential in Germany. It appears then that these contextual factors warrant an exploration of country differences in the role of family relationships in youth’s aspirations.

## The Current Study

The main contribution of this study is to systematically test the role of non‐material parental support in understanding differences in socioeconomic and immigrant background patterning in students’ aspirations. In doing so, we (a) test multiple aspects of family relationships as potential explanatory mechanisms; (b) examine the alternative roles that family relationships may play in aspirations by testing them as both mediators and moderators; and (c) explore the similarity of these mechanisms across countries. In addition, our study presents results based on European students in a research field that is dominated by US‐based studies.

**Figure 1:**
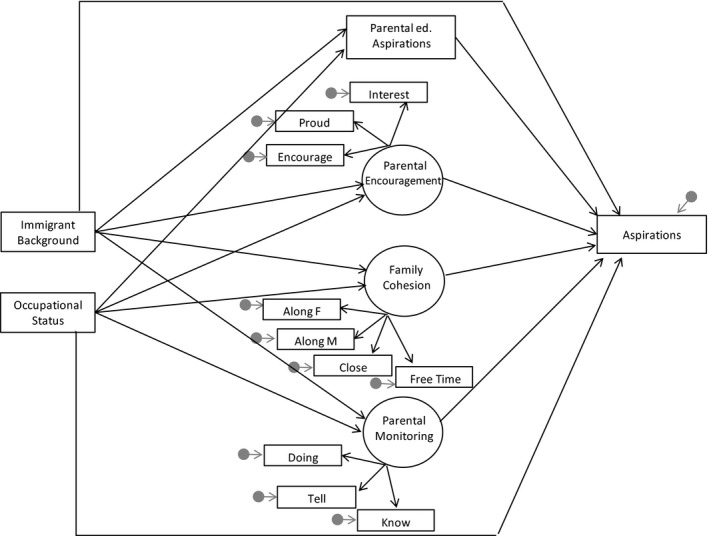
Theoretical model. *Note*. Covariates and correlations among the latent factors not shown for simplicity.

The proposed theoretical model is presented in Figure 1. We expect to find higher parental aspirations, encouragement, family cohesion, and parental monitoring among youth of immigrant and higher socioeconomic origins (Hypothesis 1). These differences in family relationships are expected to mediate the effects of socioeconomic and immigrant background on students’ occupational aspirations (Hypothesis 2). If mediation effects are observed, they have the potential to simultaneously account for the low aspirations of disadvantaged socioeconomic background children and the high aspirations of children of immigrants.

We next ask whether family relationships function differently across family background groups. If the aspirations of disadvantaged youth are more sensitive to non‐material parental support, the effect of family relationships will be stronger for students with immigrant or lower socioeconomic backgrounds (Hypothesis 3a). On the other hand, if only advantaged youth’s parents have the amount and form of human capital that can be effectively transmitted, then family relationships will be more influential for youth from the majority population or youth with a higher socioeconomic background (Hypothesis 3b).

Finally, our prime interest lies in general processes and we have no strong theoretical reason to expect meaningful differences between our countries. However, it is possible that the German tracked school system dampens the associations between family relationships and aspirations because students’ choices are already circumscribed. Alternatively, because education qualifications are particularly important in the German labor market, the associations between family relationships and aspirations may actually be stronger in Germany than Sweden. To explore these possibilities, we will analyse country moderation effects on the pathways from family background to occupational aspirations.

## Method

### Data

The data come from the first and second waves the Children of Immigrants Longitudinal Survey in Four European Countries (CILS4EU). This is a longitudinal and cross‐national study of young people in England, Germany, the Netherlands, and Sweden, funded by several European research councils (NORFACE). The project uses a two‐step cluster design: first, schools were selected, over‐sampling schools with a high proportion of immigrant youth; then two classes were randomly drawn within each school, and all students in these classes were invited to participate. The current study excludes England due to a low participation rate in the parental survey, and the Netherlands due to inconsistencies in the parental data. In Wave 1, 10,038 students 14–15 years of age in Germany and Sweden in 522 classes from 273 schools completed questionnaires and ability tests during two school hours (in the winter 2010/spring 2011 term).

The school participation rates were 75% in Sweden and 90% in Germany, and the student participation rates were 81 (Germany) and 86% (Sweden). Parents also completed questionnaires (participation rates: 59% in Sweden, 78% in Germany), and in Sweden additional information was collected from administrative registers held by Statistics Sweden (the national statistics agency). Students completed questionnaires again 1 year later (Wave 2), when most participants were in their final year of full‐time compulsory schooling. All students were informed that participation was voluntary and that their responses were anonymous. Details on the study design are described in Kalter, Jonsson, van Tubergen, and Heath (2018) and at www.cils4.eu, and the survey data are available at the GESIS data archive (www.gesis.org; ZA5353 data file).

Information on all predictors and covariates are drawn from Wave 1. Information on occupational aspirations are from 1 year later (Wave 2), which captures a period during which most participants were making important decisions about their post‐compulsory schooling choices, such as whether to continue to upper secondary school, and which program to enrol in (e.g., vocational vs. academic track, or humanities vs. natural sciences).

Although 8,254 (82%) students participated at both waves, 2,099 (25.4%) indicated that they “did not know” which occupation they would like to have (an additional *n* = 229 or 2.8% did not respond to this item). This is consistent with “don’t know” response rates reported in other studies of adolescent occupational aspirations (e.g., 27% in Australian Institute of Family Studies, 2017). Participants who did not nominate an occupation were more likely to have parents with higher occupational status and to reside in Sweden. These participants were removed from further analyses, reducing the analysis sample to *n* = 5,926 (59% of the Wave 1 sample).

### Measures

#### Outcome Variable

##### Occupational aspirations

Participants were asked “What occupation would you like to have as an adult?”. Their nomination was coded according to the 2008 International Standard Classification of Occupations (ISCO‐08), and then converted into the ISEI‐08 scale of occupational status (Ganzeboom, 2010). The ISEI is an internationally standardized scale that assigns socioeconomic status values to occupations according to their ability to mediate the association between education and income. Values range from 11 to 89, with higher values representing a higher status occupation.

#### Family Background

##### Parental occupational status

Occupational status was used as a proxy for students’ socioeconomic background because it is easily comparable across countries and is a more valid measure than household income or parental education due to substantially fewer missing data and more accurate reporting. Information was based on participants’ descriptions of each of their parents’ occupations. If a parent was not currently working, students were instructed to list the most recent occupation. Responses were, just as for children’s aspirations, coded to ISEI‐08. This score was then summed and converted to a within‐country percentile ranking.

##### Immigrant background

Immigrant background was based on students’ responses about their own as well as their parents’ country of birth and complemented with parent‐reports in the case of student non‐response. The majority population (which we use as a reference category) include those born (or adopted) in the host country, who have no parent born abroad (in cases where we have information on only one parent, immigrant background is defined according to that parent); all others are defined as having immigrant background. Students with immigrant parents from different countries are assigned their mother’s country of birth. To capture immigrant heterogeneity, a three‐category indicator of immigrant background was generated, representing: *Majority*; *European/Western background*; *non‐European/Western background*. The latter two categories distinguish between regions that are geographically, socioeconomically, and culturally closer versus distant to the host countries, which are relevant to adolescents’ integration, acculturation, and discrimination (e.g., Kalter et al., 2018). On the other hand, immigrants from non‐European/Western regions have typically overcome larger and more obstacles to migrate than those from European/Western regions, and may therefore be positively selected on various characteristics such as resilience and motivation, or educational attainment (Feliciano, 2005; Ichou, 2014).

#### Family Relationships

##### Parental educational involvement

We operationalize parental educational involvement through two measures reflecting academic socialization: *parental encouragement* and *parental aspirations*.

##### Parental encouragement

Parental encouragement reflected the interest, praise, and encouragement to put in effort and do well in school that parents convey to their child. This aligns with the definition of parental encouragement and support presented in Boonk et al.’s (2018) review of parental involvement. Students indicated the extent to which they agreed with the following three statements: “My parents … show a lot of interest in my grades and achievement in school”, “tell me that they are proud of me when I do well in school”, and “encourage me to work hard for schoo”. Response options were along a 5‐point scale ranging from *strongly disagree* to *strongly agree* (Cronbach’s α = .75).

##### Parental aspirations

Students’ parents indicated the highest level of education they wish their child to attain, ranging from minimum compulsory schooling to university. The specific level of attainment respondents could select was consistent with each country’s educational system. Responses were recoded to represent years of education and were then centered around the country mean. Parent‐reports were used to avoid potential common method bias that would arise from using student‐reports of parental aspirations. Parental aspirations and expectations are often used interchangeably in the literature. We chose to use parental aspirations rather than expectations to better capture parental values and to minimize endogeneity with the outcome variable.

##### Family cohesion

Students responded to four statements that described the social atmosphere in their family: “We like to spend free time with each other”, “we feel very close to each other”, and “how well do you get along with your mother/father?”. The first two statements had four response options: *never, sometimes, often*, and *always*, while response options for the latter two included *not well at all, not that well, well*, and *very well* (Cronbach’s α = .74).

##### Parental monitoring

Participants responded to three statements: My parents … “say that I must tell them everything that I do”, “want to know the parents of the people I hang out with”, and “I always need to tell my parents exactly where I am and what I am doing when I am not at home”. Response options were along a 5‐point scale ranging from *strongly disagree* to *strongly agree* (Cronbach’s α = .68). These items were worded to intentionally avoid measuring participants’ parental disclosure (see Stattin & Kerr, 2000).

#### Control Variables

##### Gender

Gender is based on students’ self‐reports (0 = *male*, 1 = *female*).

##### Year of birth

Year of birth ranges from 1992 to 1997, with a strong modal value of 1996.

##### Family structure

Participants indicated if they lived with both their biological (or adoptive) parents or not (0 = *not intact*, 1 = *intact family*).

##### Age of immigration

This variable represented the age that participants with an immigrant background moved to the country of residence, divided into four categories: *Born in host country* (majority or second‐generation immigrant), *arrival before 6 years of age*, *arrival between 6 and 10 years of age,* and *arrival after 10 years of age*. The latter two categories correspond approximately to the starting ages for junior and middle school, respectively.

##### Cognitive and language ability

Cognitive ability was measured using a timed pattern recognition test, considered the most culturally independent cognitive test (see Weiss, 2006). Language ability was assessed by a timed word test (synonyms or antonyms). The test scores were centered around the country mean. We controlled for test results because aspirations and family relationships are best evaluated net of the preconditions for opportunities. The puzzle with the high aspirations of children of immigrants is defined as the propensity of making the transition to higher education given their previous performance. At the time of data collection, Swedish students had never received school grades and so test scores were essential for controlling for academic ability.

##### Country and educational track

Participants’ country of residence and educational track was accounted for using a three‐category measure representing Sweden (reference category), Germany‐vocational/lower track, or Germany‐academic track.

#### Robustness Tests

As a robustness check, we used *educational* aspirations as an alternative outcome because many participants who were excluded from the main analyses due to “don’t know” responses were retained in the tests of educational aspirations. Participants were asked “What is the highest level of education you wish to get?” and could select a level of attainment ranging from minimum compulsory schooling to university, corresponding with the country’s educational system. Responses were recoded to represent years of education and were then centered around the country mean. Although only 6% of participants selected “don’t know” on the educational aspiration item, the distribution of the remaining responses varied substantially between countries. Educational aspirations were heavily skewed toward university education in Sweden (82%) compared to Germany (41%). In Sweden, this measure had limited variation as 99% of participants nominated at least upper secondary school. Thus, occupational rather than education aspirations were used as the main outcome variable.

Another robustness test controlled for additional indicators of family relationships available in our data to gauge the extent to which omitted measures may influence the results. These reflected to what extent students experience warm parenting (four items), family tensions (three items), harsh parenting (three items), and confide in their parents (two items).

### Missing Data and Analytical Strategy

Of the analysis sample, 54% of participants had information missing on at least one exogenous variable or family relationships indicator. However, only 23% were missing information on more than one variable. To maximize the sample size and retain statistical power, missing data were multiply imputed using chained equations, creating five imputed data sets in Stata 14 (Stata Corporation, 2015). Table 1 shows the missing data frequencies.

**Table 1:** Descriptives (*n* = 5,926, Unweighted): Percentage (%), Mean (*M*), and Standard Deviation (*SD*) of All Variables

	Germany	Sweden	Total sample	% missing
*n*	3,112	2,814		
Occupational aspirations, *M* (*SD*)	53.37 (21.38)	63.14 (20.56)	58.01 (21.55)	—
Family background				
Immigrant background, %				1.67
Majority	54.77	56.54	55.61	
European	18.48	16.98	17.77	
Non‐European	26.75	26.48	26.63	
Parental occupational status, *M* (*SD*)	0.50 (0.29)	0.49 (0.28)	0.49 (0.28)	15.81/16.05^a^
Family relationships				
Parental aspirations^b^, *M* (*SD*)	12.56 (2.55)	15.66 (1.15)	13.81 (2.59)	29.06
Parental encouragement^c^, *M* (*SD*)	4.22 (0.69)	4.50 (0.61)	4.35 (0.67)	0.46–0.51
Family cohesion^c^, *M* (*SD*)	3.08 (0.57)	3.32 (0.57)	3.20 (0.58)	4.93–12.22
Parental monitoring^c^, *M* (*SD*)	3.11 (0.92)	2.89 (0.86)	3.00 (0.90)	4.94–5.11
Control variables				
Gender, %				< 1
Male	50.78	49.27	50.06	
Female	49.22	50.73	49.94	
Age of immigration, %				—
Born in host country	90.13	87.92	89.08	
Before 6 years of age	5.27	3.55	4.45	
6–10 years of age	2.83	4.76	3.75	
Above 10 years of age	1.77	3.77	2.72	
Family structure, %				4.74
Not intact	31.66	31.33	31.50	
Intact	68.34	68.67	68.50	
Year of birth, *M* (*SD*)	1995 (0.75)	1996 (0.27)	1996 (0.69)	1.28
Language ability^b^, *M* (*SD*)	11.41 (4.44)	18.34 (4.99)	14.64 (5.83)	1.92
Cognitive ability^b^, *M* (*SD*)	19.09 (4.00)	17.55 (4.72)	18.38 (4.41)	2.16
Country + tracking, %				—
Sweden	—	—	47.49	
Vocational	80.59	—	42.32	
Academic	19.41	—	10.19	

Note

Parental occupational status = *ISEI rank*.

^a^ % missing for mothers/fathers.

^b^ Presents values before within‐country centering.

^c^ Summarizes indicators for latent factors.

Structural Equation Modeling was performed in Mplus 7 (Muthén & Muthén, 1998–2012) to test the pathways from family background to occupational aspirations. SEM was used because it permits the modeling of latent constructs and simultaneous testing of direct and indirect effects. Maximum likelihood estimation with robust standard errors was used to account for non‐normality of the latent factor indicators.

A two‐step model‐building process was followed: first, establishing the measurement model representing family relationships, and then testing the structural model. Analyses controlled for the clustering of students within classrooms to ensure that standard errors were not underestimated. Survey weights were also used to adjust for the oversampling of immigrant‐dense schools and ensure that the samples were nationally representative and to give each country equal weight. In the tests of structural paths, gender, age, country, tracking, age of immigration, family structure, and cognitive and language test results were included as covariates of occupational aspirations and family relationships. The chi‐square test statistic, the comparative fit index (CFI) and the root mean square error of approximation (RMSEA) were used to evaluate model fit. CFI values above .90 and .95 were considered to reflect acceptable and excellent fit, respectively, while a RMSEA below .06 indicated acceptable model fit (Hu & Bentler, 1999; Marsh, Hau, & Wen, 2004).

Multigroup Confirmatory Factor Analyses were performed to assess measurement invariance and moderation effects across the family background categories and countries. Measurement invariance confirms if the factor structure and latent factor indicators function similarly across groups. Support for measurement invariance is accepted if model fit does not decrease significantly as equality constraints across groups are imposed on parameters of the model (see Meade & Lautenschlager, 2004). As chi‐square tends to be oversensitive in large samples (Cheung & Rensvold, 2002), changes in CFI > .002 were also used to indicate non‐invariance in the measurement and structural models (Meade, Johnson, & Braddy, 2008). A dichotomous measure of parental occupational status was generated for the multiple group analyses. To ensure these categories were distinct from immigrant background, this measure distinguished between parental occupational status in the highest quartile compared to lower percentiles.

## Results

### Sample Descriptives and Mean Occupational Aspirations

The unweighted sample descriptive statistics before multiple imputation are presented in Table 1. Around 27% of the sample had a non‐European background and 18% had a European background. Higher average occupational aspirations were observed among students with a European immigrant background (*M* = 60.42, 95% CI [58.60, 62.25]), and especially those with a non‐European background (*M* = 64.12, 95% CI [62.36, 65.88]), compared to the majority population (*M* = 55.94, 95% CI [54.92, 56.96]). Higher average aspirations were also found among students with high parental occupational status (*M* = 64.63, 95% CI [62.98, 66.27]) than those with lower parental occupational status (*M* = 54.58, 95% CI [53.60, 55.57]).

### Predicting Students’ Aspirations

The measurement model showed excellent model fit for the three latent factors representing family relationships (χ^2^ = 145.58, *df* = 31, *p* < .05; CFI = .976; RMSEA = .025), with factor loadings ranging from .50 to .74. Measurement invariance was observed across immigrant groups, across students with lower versus high parental occupational status and across countries (see the Appendix S1 for details of model fit and measurement invariance). Bivariate associations among the family background variables, family relationships, and occupational aspirations are presented in Table 2. Positive correlations were observed among family relationships and occupational aspirations, with parental aspirations showing the strongest correlation with youth’s occupational aspirations (*r* = .41), followed by family cohesion (*r* = .16) and parental encouragement (*r* = .15). Higher parental occupational status and immigrant background were each positively associated with higher parental aspirations, parental encouragement, family cohesion, parental monitoring and occupational aspirations.

**Table 2:** Bivariate Correlations Among Family Background, Family Relationships, and Occupational Aspirations (Weighted)

	Occupational aspirations	Parental aspirations	Parental encouragement	Family cohesion	Parental monitoring
Parental aspirations	.41				
Parental encouragement	.15	.12			
Family cohesion	.16	.13	.67		
Parental monitoring	.05	.09	.11	−.06	
Parental occupational status (ISEI rank)	.27	.27	.07	.08	.07
European background^a^	.21	.32	.14	.10	.01^*^
Non‐European background^a^	.38	.46	.32	.24	.38

Note

All coefficients *p* < .05, except *p .05.

^a^ Bivariate standardized regression coefficients shown, using majority youth as reference category.

**Figure 2:**
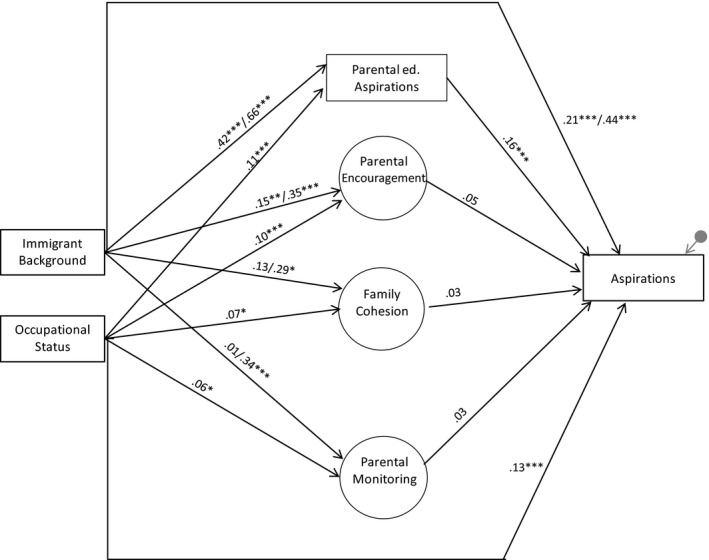
Estimates for the hypothesised structural paths. *p < .05; **p < .01; ***p < .001. *Note*. Immigrant background coefficients = European/Non‐European background. Results for covariates, factor loadings and correlations not shown for simplicity (see Appendix S1 for details).

The structural model was then tested to investigate the mutually adjusted estimates in the pathways from family background to students’ occupational aspirations (Figure 2). This model showed acceptable fit to the data (χ^2^ = 781.36, *df* = 136 *p* < .05; CFI = .93; RMSEA = .028) and key structural paths are shown in Figure 2 (see the Appendix S1 for estimates for the covariates). As expected, students with an immigrant background were more likely to have greater parental aspirations, encouragement, family cohesion and parental monitoring than majority students. This was particularly clear for students with a non‐European background whose family relationship scores were between .29 and .66 of a standard deviation higher than majority youth, which are non‐trivial effects. Higher parental occupational status also predicted stronger parental aspirations, encouragement, family cohesion, and parental monitoring. A one standard deviation increase in parental occupational status percentile was associated with .11 of a standard deviation increase in parental aspirations and .06–.10 of a standard deviation increase in parental encouragement, family cohesion and parental monitoring.

Immigrant background and higher parental occupational status were also each directly associated with higher occupational aspirations. After controlling for family relationships, parental occupational status and all covariates, the occupational aspirations of European and non‐European immigrants were .21 and .44 standard deviations higher than students of majority origin, respectively, which are substantial net effects. As expected, higher parental aspirations predicted higher occupational aspirations 1 year later, with a one standard deviation increase in parental educational aspirations corresponding to .16 of a standard deviation increase in students’ occupational aspirations. However, unexpectedly, none of the family relationship latent factors showed significant associations with occupational aspirations. The model accounted for 31% of the variance in youth’s occupational aspirations.

### Mediating Effects

We next examined the indirect effects of family background on occupational aspirations. Significant mediating effects for parental aspirations were observed but not for parental encouragement, family cohesion, or parental monitoring. Table 3 presents the mediation results for parental aspirations. Here, we see that parental aspirations accounted for 23% and 18% of the effects for European and non‐European immigrant background, respectively (i.e., indirect effect/total effect). Parental aspirations mediated 11% of the effect of parental occupational status on students’ occupational aspirations.

**Table 3:** Mediation Effects of Parental Aspirations

Family background predictor	Indirect effect	Total indirect effect	Total effect	% mediated
European background	.066***	.077***	.289**	22.83%
Non‐European background	.105**	.140***	.581**	18.07%
Parental occupational status (ISEI rank)	.018***	.027***	.161***	11.18%

**p < .01; ***p < .001

As to our main question, Table 3 reveals that although parental aspirations mediate some of the immigrant background effect, overall, the mechanism of family relationships did not explain a substantial proportion of the association with students’ aspirations. Thus, even net of family relationships children of immigrants have substantially higher aspirations than majority students. The low aspirations of students with a less, as compared to a more advantaged socioeconomic background did not appear to strongly depend on family relationships either.

### Moderation Effects

Multigroup analyses were then performed to examine moderating effects of immigrant background and parental occupational status on the influence of family relationships on occupational aspirations. However, we found no statistically significant interactions for parental occupational status (Satorra–Bentler Δχ^2^ = 10.93, *df* = 4, *p* = .03; ΔCFI = .001) or immigrant background (Satorra–Bentler Δχ^2^ = 2.78, *df* = 8, *p* = .95; ΔCFI = .001) with any of the family relationship latent factors or with parental aspirations. A sensitivity test that used the continuous measure of parental occupational status also found no significant interactions.

However, some moderating effects of country on the structural paths were observed (Satorra–Bentler Δχ^2^ = 115.52, *df* = 19, *p* = < .000; ΔCFI = .01). The estimates for parental aspirations on student aspirations were slightly stronger in Germany than Sweden (Germany β = .18, *SE* = .03; Sweden β = .12, *SE* = .03). The effect of immigrant background on parental aspirations was also stronger in Germany (European β = .48, *SE* = .07; Non‐European β = .80, *SE* = .09) compared to Sweden (European β = .24, *SE* = .08; Non‐European β = .35, *SE* = .09). This may partly be understood against the larger variance in parental aspirations in Germany, probably due to the circumscribed opportunities for students in lower tracks. In accordance with these differences, parental aspirations mediated a greater proportion of immigrant background effects in Germany (European = 29.04%; Non‐European = 30.77%) than in Sweden (European = 10.03%; Non‐European = 5.78%).

### Robustness Tests

Our preference of using occupational nominations as an indicator of aspirations, while theoretically reasonable, also resulted in a reduced sample size. To test the robustness of the key findings, the analyses were re‐run using educational aspirations as an alternative outcome. The results (see Appendix S1), came to substantively similar results to those using occupational aspirations, indicating little influence of the latent factors. This suggests that our results can be interpreted as a general representation of aspirations. In addition, sensitivity tests that expanded the socioeconomic variable with multiple indicators (household income, parental education, and employment) did not change the findings (see Appendix S1).

Our preferred model included three dimensions of family relationships, but an argument can be made for the importance of other types. We performed exploratory factor analyses that included additional items reflecting parental warmth, confiding in parents, family tension, and harsh parenting. These showed that the current three‐factor model produced the clearest factor structure without compromising the theoretical framework or empirical validity of the factors. Nevertheless, we also tested two ordinary least squares regression models: the first including our three dimensions of interest, and the second adding all additional indicators of family relationships. Adding these other dimensions of family relationships raised issues of multicollinearity while increasing the explained variance of occupational aspirations by only 1%. We chose therefore to present the more parsimonious three‐factor model.

## Discussion

We addressed the question of whether and to what extent family relationships—understood as parental aspirations, encouragement, family cohesion, and parental monitoring—could account for sociodemographic differences in young people’s aspirations. We were particularly interested in whether these aspects of non‐material parental support could resolve the puzzle that our two indicators of family background—low socioeconomic origin and immigrant background—could have such disparate associations with youth’s aspirations. To test hypotheses regarding the role of family relationships in occupational aspirations, we applied SEM to cross‐national data (CILS4EU) comprising around 6,000 secondary‐school students in Germany and Sweden.

We set the scene by confirming empirically the expected relations between socioeconomic origin and students’ aspirations (Jackson, 2013), and between immigrant origin and students' aspirations (cf. Heath & Brinbaum, 2014; Jackson et al., 2012). It is noteworthy that students with a non‐European immigrant background had occupational aspirations that were more than one third of a standard deviation higher than those of the majority population. The socioeconomic and immigrant gradients in students’ aspirations were also observed in the full SEM model that mutually adjusted for both aspects of family background as well as family relationships, ability tests, and exogenous factors. The estimates for immigrant background were even stronger in the full model, with the aspirations of non‐European immigrants being nearly half a standard deviation higher than those of majority youth. When we instead used educational aspirations as the outcome, the results were very similar, and expanding the indicators of socioeconomic background led to the same results.

### Mediating Effects of Family Relationships

In line with Hypothesis 1, we found that children of immigrant (particularly non‐European) background and those who are socioeconomically advantaged had stronger family relationships in terms of parental aspirations, encouragement, family cohesion, and parental monitoring. Parental aspirations, in turn, significantly predicted higher aspirations, but the other aspects of family relationships did not. Parental aspirations mediated 11%–23% of the effects of family background on aspirations but no mediation effects were observed for parental encouragement, family cohesion, or parental monitoring, meaning that Hypothesis 2 found mixed support. The mediation effects were similar to those reported in other studies on parental aspirations and student outcomes (e.g., von Otter, 2014) but also consistent with others finding no mediating effects for subtypes of parental involvement (Carolan & Wasserman, 2015). We believe that the nature of our measures, range of controls, and the longitudinal design were effective in protecting against confounding that may otherwise result in overestimated associations. An additional 4%–6% of family background effects were mediated through parental encouragement, family cohesion, and parental monitoring (albeit not statistically significantly). When we controlled for additional aspects of family relationships available in our data, over 99% of the family background‐aspiration associations remained. Therefore, even if measurement error and model mis‐specification might lead to underestimations of the mediating role of non‐material parental support, it appears unlikely that the conundrum of the high aspirations of children of immigrants could be more than partly resolved by alluding to family relationships.

### Moderation Effects

We further examined the role of family relationships, by analysing whether non‐material parental support might be more “effective” for some sociodemographic groups, which would impact on the equalizing possibilities of family relationships. However, no significant interactions between family background and family relationships were found, and thus neither Hypothesis 3a, nor 3b were supported. This contrasts with Coleman’s (1988) assumption that strong family relationships are necessary for the transmission of human capital, but our results are in line with the results of some previous studies (e.g., Jeynes, 2007; Ream & Palardy, 2008; Wang & Sheikh‐Khalil, 2014).

Finally, we asked whether stronger family relationships were more effective in Sweden, with a comprehensive educational system, than in Germany, with a tracked system. However, the link between parental aspirations and student aspirations was slightly stronger in Germany than in Sweden. Also, in Germany immigrant parents held higher aspirations than the majority population to a greater extent than in Sweden, leading to a larger share of the immigrant effect being mediated by parental aspirations. Future studies should replicate these country differences and investigate if they stem from differences in the role of credentials in the labor market or between types of educational tracks (i.e., vocational or academic).

### Limitations

Despite several unique advantages—including the large and nationally representative samples of students in two countries, longitudinal design, multiple aspects of family relationships, comprehensive control variables, and analysing socioeconomic and immigrant background simultaneously—this study also knows some limitations. While we measured family relationships 1 year prior to assessing participants’ occupational aspirations, identifying causality between these processes is cumbersome. It is likely that parental aspirations share reciprocal associations with students’ own ability and ambitions, and we accounted for this possibility as best we could by controlling for ability tests. Although self‐reported school grades were available for Germany, the analyses controlled for cognitive and language ability instead due to their protection against common method bias, conflation with students’ aspirations and comparability across countries and school systems.

Also, the theoretical model is built on assumptions that unobservables do not bias our results. Most of the plausible confounders would lead to an attenuation of the associations involving family relationships. However, after controlling for a large range of confounders we observed that the associations between family relationships and student aspirations were mostly quite weak (cf. Benner et al., 2016). Although a stronger effect was observed for parental aspirations, it should be considered cautiously in terms of a causal interpretation because it is may be upwardly biased. It should be noted that these results apply to students’ aspirations, and it is possible that tests on other outcomes, such as educational achievement may arrive at different conclusions.

### Directions for Future Research and Conclusion

Our results, suggesting that family relationships are not overly efficient in converting parental resources into filial aspirations, make it natural to ask what it is then, that explains why lower socioeconomic status predicts relatively low aspirations, but immigrant background predicts very high aspirations. An unknown part will be the genetic parent–child transmission of abilities and personality traits that shape aspirations, but a perhaps more likely candidate is the long‐term day‐to‐day socialization processes as suggested by the results for parental aspirations (cf. Jeynes, 2018). It is possible that other non‐material means than those we have measured play a role. For example, as education‐related values are often domain specific (Guay & Bureau, 2018), it is of interest to investigate to what extent discipline‐specific parental involvement relates to family background differences in students’ aspirations (e.g., Arens & Jude, 2017).

The socioeconomic gradient, aside from the typical social and economic mechanisms, could still depend on subtle family processes. For example, social position theory refers to the generalized desire to avoid downward mobility between generations (Boudon, 1974; Erikson & Jonsson, 1996), a desire that could theoretically be internalized in children through subtle parent–child processes related to expectations (Jeynes, 2018), cultural habits (Lareau, 2011) or social modeling, which are all difficult to observe in large‐scale data. However, the only way of applying the immigrant effect to this narrative is to refer to parents’ position in their country of origin (Engzell, 2019), but it appears unlikely that this would account for the large aspirational advantage that we found for children of non‐European background. Other potential explanations are that children of immigrant background are “over‐advised” in schools (van de Werfhorst & van Tubergen, 2007) that they underestimate the demands at higher levels of education (Dollmann & Weissman, 2019), or that they believe a high education helps to protect against discrimination in the labor market (e.g., Jonsson & Rudolphi, 2011).

It appears, then, that the most challenging result to explain is the very high aspirations among children of immigrant origin, even when we take their stronger family relationships into account. Aspiration trajectories of children of immigrants should be tested across time, to understand aspiration‐expectation discrepancies as students’ progress through the educational system and reach their eventual educational attainment. To arrive at more robust causal estimates such “life‐course” longitudinal approaches would be valuable, as would studies with an experimental or quasi‐experimental design. More research on this topic would help us to better understand how aspirations can be upheld also when opportunities do not abound.

## Supporting information


**Appendix S1.** Additional information on model fit, estimates for covariates, and robustness tests.
