# Supplementary material for: Students’ Occupational Aspirations: Can Family Relationships Account for Differences Between Immigrant and Socioeconomic Groups?
Source: Child Dev. 2020 Jun 23;92(1):157–73. doi: 10.1111/cdev.13378 (PMC7891578; doi:10.1111/cdev.13378)
Supplement: Supplementary file 1 — Appendix S1. Additional information on model fit, estimates for covariates, and robustness tests. [file CDEV-92-157-s001.doc]

**Appendix**

Table A1. Measurement invariance across immigrant, occupational status and country groups

| Grouping characteristics |  | X^2^ | df | CLI | RSMEA | 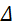 X^2^ | 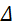 TLI |
| --- | --- | --- | --- | --- | --- | --- | --- |
| Immigrant background | Majority | 95.99 | 31 | .978 | .025 |  |  |
|  | European | 69.62 | 31 | .971 | .034 |  |  |
|  | Non-European | 94.72 | 31 | .945 | .036 |  |  |
|  | No constraints | 263.70 | 93 | .968 | .030 |  |  |
|  | Metric invariance | 276.74 | 107 | .967 | .030 | *p* > .05 | .001 |
|  | Scalar invariance | 300.06 | 121 | .967 | .027 | *p* > .05 | .001 |
|  |  |  |  |  |  |  |  |
| Parental occupational status | Lower occupational status | 124.98 | 31 | .974 | .026 |  |  |
|  | High occupational status | 59.01 | 31 | .979 | .025 |  |  |
|  | No constraints | 190.89 | 62 | .975 | .026 |  |  |
|  | Metric invariance | 204.00 | 69 | .974 | .026 | *p* > .05 | .001 |
|  | Scalar invariance | 224.85 | 79 | .972 | .025 | *p* < .05 | .002 |
|  |  |  |  |  |  |  |  |
| Country | Sweden | 145.61 | 31 | .970 | .036 |  |  |
|  | Germany | 67.98 | 31 | .982 | .020 |  |  |
|  | No constraints | 194.86 | 62 | .976 | .027 |  |  |
|  | Metric invariance | 203.63 | 69 | .976 | .026 | *p* < .05 | .000 |
|  | Scalar invariance | 561.03 | .76 | .912 | .046 | *p* > .05 | .064 |
|  | Partial scalar invariance | 208.93 | 71 | .975 | .026 | *p* < .05 | .001 |

Table A2. Estimates for all covariates

|  | Predictor | Outcome | |  |  |  |
| --- | --- | --- | --- | --- | --- | --- |
|  | | Parental  aspirations | Parental  encouragement | Family  cohesion | Parental  monitoring | Students occ.  aspirations |
|  | *Gender* |  |  |  |  |  |
|  | Male | Ref. | Ref. | Ref. | Ref. | Ref. |
|  | Female | .09 (.04)* | -.08 (.05) | -.15 (.06)** | .19 (.04)*** | .27 (.03)*** |
|  | *Year of birth ^a^* | .07 (.02)*** | .05 (.03)** | .07 (.03) | .07 (.03)* | .03 (.02) |
|  | *Age of immigration ^a^* |  |  |  |  |  |
|  | Born in host country | Ref. | Ref. | Ref. | Ref. | Ref. |
|  | Before 6 years of age | .17 (.13) | .10 (.10) | -.09 (.13) | .12 (.12) | .07 (.08) |
|  | 6-10 years of age | .07 (.17) | .01 (.12) | -.05 (.14) | -.17 (.12) | .16 (.11) |
|  | Above 10 years of age | .50 (.14)*** | -.18 (.15) | .35 (.15)* | .09 (.12) | .23 (.10)* |
|  | *Language ability ^a^* | .10 (.02)*** | -.01 (.03) | .00 (.02) | -.09 (.03)** | .08 (.02)*** |
|  | *Cognitive ability ^a^* | .09 (.02)*** | -.03 (.02) | .00 (.02) | -.03 (.03) | .14 (.02)*** |
|  | *Family structure* |  |  |  |  |  |
|  | Not intact | Ref. | Ref. | Ref. | Ref. | Ref. |
|  | Intact | .05 (.04) | .17 (.05)** | .32 (.06)*** | .11 (.05)* | .01 (.03) |
|  | *Country+track* |  |  |  |  |  |
|  | Sweden | Ref. | Ref. | Ref. | Ref. | Ref. |
|  | Germany vocational | -.35 (.05)*** | -.43 (.05)*** | -.46 (.06)*** | .41 (.07)*** | -.45 (.04)*** |
|  | Germany academic | .78 (.05)*** | -.43 (.08)*** | -.26 (.09)** | .47 (.07)*** | .11 (.06)* |

*Note.* *** *p* < .001; ** *p* < .01; * *p* < .05; Standardised coefficients and standard errors presented.

Figure A1. Results for key structural paths predicting youth’s *educational* aspirations, *n* = 7,742. Estimates for covariates and factor loadings not shown for simplicity.

Table A3. Mediation effects of parental educational aspirations on youth’s *educational* aspirations

| Social origin predictor | Indirect effect | Total indirect effect | Total effect | Proportion mediated |
| --- | --- | --- | --- | --- |
| European | .099** | .109*** | .265*** | 37.36% |
| Non-European | .165** | .185*** | .469*** | 34.18% |
| Parental occupational status | .031*** | .038*** | .126*** | 24.60% |

*Note.* Standardised coefficients; *** *p* < .001; ** *p* < .01; * *p* < .05.

Figure A2. Results for key structural paths using a composite measure of SES, *n* = 5,926. Estimates for covariates and factor loadings not shown for simplicity.
